# Subxiphoid pericardial drainage for gastric tube ulcer penetrating the pericardium after esophagectomy: A case report

**DOI:** 10.1016/j.ijscr.2024.109260

**Published:** 2024-01-13

**Authors:** Shinya Ohno, Yoshihiro Tanaka, Yuta Sato, Takayoshi Kato, Kiyoshi Doi, Nobuhisa Matsuhashi

**Affiliations:** a Department of Gastroenterological Surgery, Pediatric Surgery, Gifu Graduate School of Medicine, Gifu, Japan; b Department of General and Cardiothoracic Surgery, Graduate School of Medicine, Gifu University, Gifu, Japan

**Keywords:** Esophageal cancer, Surgical drainage, Continuous irrigational lavage, Posterior mediastinal route

## Abstract

**Introduction:**

Reconstructed gastric tube ulcers are common complications of esophagectomy. When the pericardium is penetrated, digestive juices can cause severe cardiac inflammation, leading to an extremely poor prognosis. We report the first case of pericardial penetration of a constructed stomach tube via the posterior mediastinal route and the first use of subxiphoid pericardial drainage and continuous irrigation lavage.

**Presentation of case:**

This case involved a 50-year-old woman who underwent an esophagectomy for esophageal cancer nine years prior with gastric tube reconstruction via the posterior mediastinal route. She developed pericardial penetration due to a gastric tube ulcer. Her respiratory and circulatory condition worsened, and pericardial drainage and a prophylactic tracheostomy were performed to prevent septic shock. A 5-cm longitudinal incision was made in the epigastric region, and a 4-cm T-shaped incision was made through the pericardium. Two double-lumen drainage tubes were placed in the anterior and posterior pericardium, and continuous irrigation was initiated via each tube. We successfully treated the patient without complications using subxiphoid pericardial drainage and continuous irrigation lavage, and she was discharged on postoperative day 23.

**Discussion:**

We presented this case to discuss surgical techniques and optimal treatment strategies.

**Conclusion:**

Subxiphoid pericardial drainage and continuous irrigational lavage are effective for pericardial penetration of a constructed stomach tube via the posterior mediastinal route.

## Highlights

• Reconstructed gastric tube ulcers are common complications of esophagectomy.
• Digestive juices can cause severe cardiac inflammation, with a poor prognosis.
• First case pericardial penetration of stomach tube via posterior mediastinal route.
• First use of subxiphoid pericardial drainage and continuous irrigation lavage.
• Subxiphoid drainage and continuous lavage are effective for pericardial penetration.

## 1 Introduction

When a gastric tube ulcer penetrates the mediastinum, digestive juices drain into the mediastinum, causing severe inflammation. These juices can also penetrate the pericardium, causing severe cardiac inflammation with a poor prognosis (mortality rate of 33–50 %) [1,2].

Subxiphoid pericardial drainage is used in case of pericardium tamponade [3]. Subxiphoid pericardial drainage and continuous irrigation effectively manage gastric tube penetration via the posterior mediastinal route. We describe the first case of pericardial penetration by the gastric tube via the posterior mediastinal route and the use of this management technique. This study was reported in line with the SCARE 2023 criteria [4].

## 2 Presentation of case

A 50-year-old woman underwent esophagectomy, proximal gastrectomy, and gastric tube reconstruction via the posterior mediastinal route for esophageal cancer. Chemoradiation for the mediastinal lymph nodes recurrence at 8 months after surgery achieved a complete response. Nine years later, the patient presented with chest pain. She had been taking nonsteroidal anti-inflammatory drugs but no proton pump inhibitors.

**Fig. 1:**
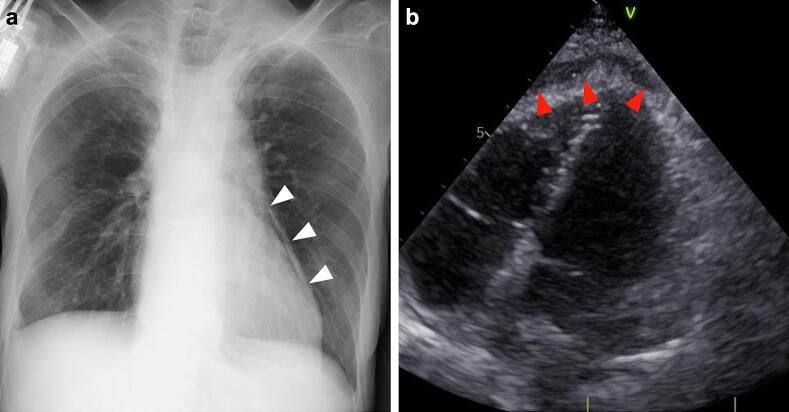
Examinations at the time of emergency. (a) Plain radiography revealed pneumopericardium (white arrowheads). (b) Echocardiography showed pericardial effusion (red arrowheads). (For interpretation of the references to colour in this figure legend, the reader is referred to the web version of this article.)

**Fig. 2:**
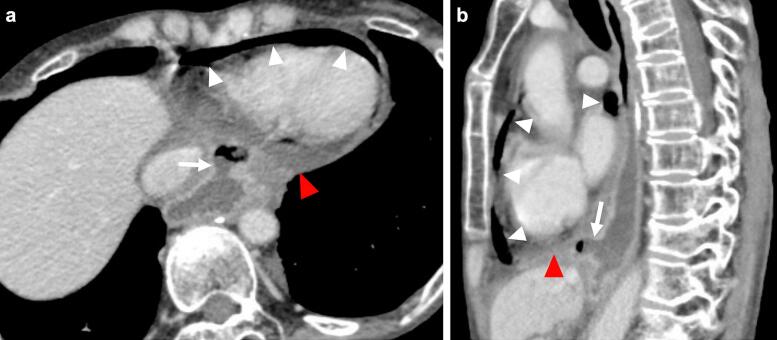
Contrast-enhanced computed tomography (CT) images. (a) The axial CT image revealed air (white arrowheads) and fluid (red arrowhead) in the pericardium, and a gastric tube ulcer toward the pericardium (white arrow). (b) The sagittal CT image revealed air (white arrowheads) and fluid (red arrowhead) in the pericardium, and a gastric tube ulcer was located on the diaphragm (white arrow). (For interpretation of the references to colour in this figure legend, the reader is referred to the web version of this article.)

Chest radiography revealed a pneumopericardium (Fig. 1A), and echocardiography revealed a pericardial effusion (Fig. 1B). Contrast-enhanced computed tomography showed air and fluid in the pericardium and a suspected ulcer in the gastric tube above the diaphragm, confirming the diagnosis (Fig. 2A, B).

**Fig. 3:**
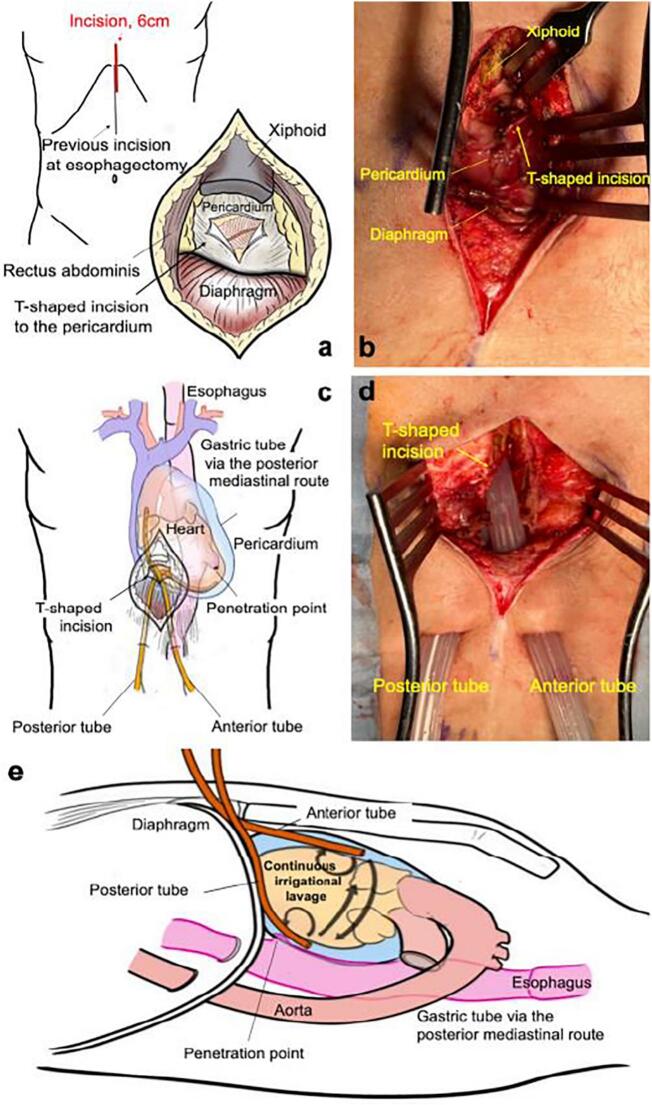
Surgical illustrations and intraoperative findings. (a) The illustration of the longitudinal incision in the epigastric region and 4-cm T-shaped incision through the pericardium. (b) The 4 cm T-shaped incision through the pericardium. (c) Illustration after the placement of drains in the anterior and posterior pericardial sac. (d) Two double-lumen tubes were placed in the pericardium through the pericardial T-shaped incision. The right drain was placed in the anterior pericardial sac, and the left was placed in the posterior pericardial sac. (e) Illustration of continuous irrigational lavage via each tube.

Because the patient's respiratory and circulatory condition were unstable, surgical pericardial drainage and prophylactic tracheostomy were performed under general intubated anesthesia. The penetration was located on the dorsal pericardium, and surgery was expected to be difficult due to adhesions, so the patient was treated with subxiphoid pericardial drainage. A 5-cm longitudinal incision was made in the epigastric region, the pericardium was reached via a subxiphoid path from the ventral side of the diaphragm, and a 4-cm T-shaped incision was made through the pericardium (Fig. 3A, B).

Two 20Fr double-lumen drainage tubes, which allowed continuous infusion of the lavage solution, were placed in the anterior and posterior pericardium (Fig. 3C, D). Continuous irrigational lavage with saline solution at 37 °C was initiated at 75 ml/h (Fig. 3E). Candida glabrata was cultured from the pericardial effusion. The patient was treated with micafungin 150 mg/day for 10 days until drainage cultures would be negative findings.

**Fig. 4:**
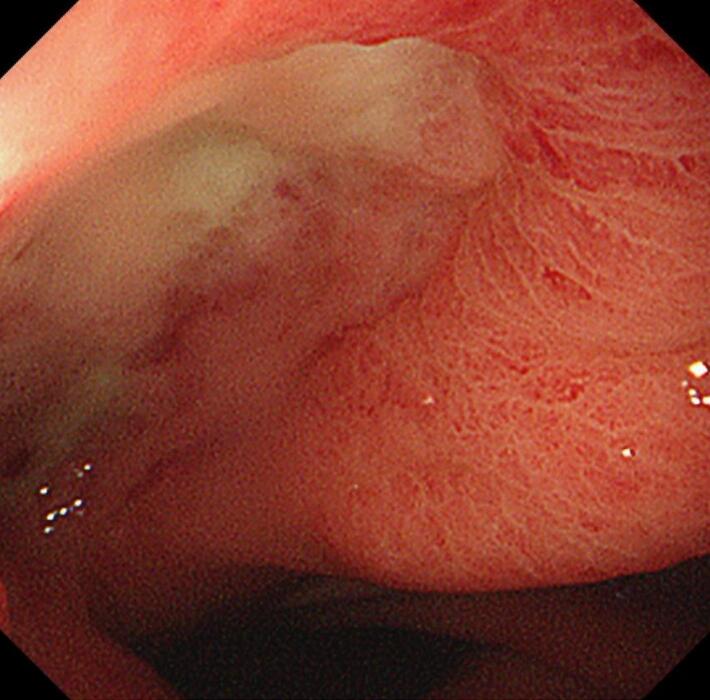
Postoperative upper endoscopy. Postoperative upper endoscopy revealed a gastric tube ulcer but no fistula or cancer recurrence.

The patient was weaned from the ventilator on postoperative day (POD) 8. On POD 13, an upper endoscopy revealed a gastric tube ulcer but no fistula or cancer recurrence (Fig. 4). On POD 17, continuous irrigation was completed, and the tubes were removed. The patient was discharged on POD 23.

## 3 Discussion

Reconstructed gastric tube ulcers have few subjective symptoms because of the sympathetic nerve transection during creation of the gastric tube [5]; however, they are often only discovered in severe cases. When a gastric tube ulcer penetrates the pericardium, most patients present with symptoms such as fever, chest pain, respiratory distress, and hematemesis [[5], [6], [7], [8]].

Reports of surgical drainage include right thoracotomy [9], left thoracoscopy [8], and median sternotomy [6], all accomplished via the retrosternal route. Surgical drainage via the posterior mediastinal route is difficult because the gastric tube is located on the dorsal side of the pericardium, adhesions from the previous operation are present in the right thoracic cavity, and the descending aorta occupies the left thoracic cavity.

In contrast, subxiphoid pericardial drainage offers the following advantages: it is less invasive than other surgical drainage techniques, the spread of surgical contamination is minimized, and if the patient's condition is good, the technique can be performed under local anesthesia within 30 min [10]. In addition, the creation of a closed space between the pericardium and the drainage tubes allows for closed lavage of the pericardium. Continuous irrigation is useful in controlling chemical inflammation and infections.

With this technique, there is no clear standard criteria for continuous intrapericardial lavage flow rate, however, we should strictly check the drainage volume per 2 h, with special attention paid to avoiding cardiac tamponade. After drainage cultures were negative and fistula closure was confirmed, we determined to cease drainage. Additional surgery may be required if the infection is uncontrolled or fistula closure does not occur.

## 4 Conclusion

Subxiphoid pericardial drainage and continuous irrigational lavage are effective for pericardial penetration of a constructed stomach tube via the posterior mediastinal route.

## Availability of data and materials

Not applicable.

## Consent for publication

Not applicable.

## Ethics approval and consent to participate

This study was approved by the review board of Gifu University Hospital (Approval no. 2019-170), and informed consent was obtained from the patient.

## Funding

None.

## Author contribution

Shinya Ohno drafted the manuscript. Shinya Ohno, Yoshihiro Tanaka, Yuta Sato, and Takayoshi Kato performed surgeries. Yoshihiro Tanaka, Yuta Sato, Kiyoshi Doi, and Nobuhisa Matsuhashi provided academic advice.

## Guarantor

Yoshihiro Tanaka.

## Conflict of interest statement

We have no conflicts of interest.
